# Antigen-Presentation Competence in Gynecologic Cancers: Mechanisms, Evidence and Translational Priorities

**DOI:** 10.3390/cells15191809

**Published:** 2026-10-03

**Authors:** Zihao Xu, Yifan Liu, Jun Xu, Mengxin Xiong

**Affiliations:** 1 School of Chinese Medicine, Hubei University of Chinese Medicine, Wuhan 430065, China; 2527012087@stmail.hbucm.edu.cn (Z.X.); 2232701111@stmail.hbucm.edu.cn (Y.L.); 2 School of Basic Medical Sciences, Hubei University of Chinese Medicine, Wuhan 430065, China; 3 The First Clinical Medical School, Hubei University of Chinese Medicine, Wuhan 430065, China

**Keywords:** antigen presentation, gynecologic cancer, HLA class I, immunopeptidomics, cDC1, immune-checkpoint inhibition, biomarker, immune escape

## Abstract

Immune-checkpoint biomarkers in gynecologic cancers primarily estimate antigenic potential or characterize an inhibited immune response. However, they do not establish whether malignant cells are visible to antigen-specific lymphocytes. We define antigen-presentation competence as the capacity of the tumor–immune ecosystem to generate antigenic peptides, display them through functional human leukocyte antigen molecules, prime cognate T cells through professional antigen-presenting cells, and permit recognition of malignant cells by effector lymphocytes. Recurrent defects have been reported at each step in cervical, endometrial, and ovarian cancers, but the evidence is not equally mature across these diseases. Cervical studies emphasize human papillomavirus-associated immune selection and antigen-processing machinery abnormalities; endometrial cancer provides relatively direct treatment-linked evidence for major histocompatibility complex class I and interferon-pathway defects; and ovarian cancer offers direct immunopeptidomic and multiregion evidence that antigen availability, presentation, and immune recognition can diverge. Taken together, the literature makes antigen-presentation competence a testable functional framework. It does not yet justify a biomarker or clinical score. Prospective work will need spatial and longitudinal measurements of tumor presentation, dendritic-cell priming, and T-cell recognition. A key test is whether these measurements improve prediction beyond mismatch repair, polymerase epsilon, programmed death-ligand 1, and tumor mutational burden.

## 1. Introduction

Immune-checkpoint inhibition has improved outcomes in gynecologic cancers, but durable benefit remains uneven within and across disease groups. Molecularly selected endometrial cancers can be highly responsive [1,2], whereas activity in unselected cervical and ovarian cancers is more variable. Current stratification relies principally on mismatch-repair deficiency (dMMR) or microsatellite instability (MSI), pathogenic DNA polymerase epsilon (POLE) alterations, programmed death-ligand 1 (PD-L1), and tumor mutational burden (TMB). These markers are clinically useful, yet they interrogate different and incomplete aspects of antitumor immunity. dMMR, POLE mutation, and TMB estimate the opportunity to generate altered peptides; PD-L1 can indicate checkpoint activity or an interferon-associated adaptive response. None establishes whether relevant peptides reach the tumor-cell surface or whether cognate T cells are generated and can recognize them [3,4].

Antigen processing and presentation comprise a series of linked gates rather than a single biomarker axis [5]. Intracellular proteins must yield suitable peptides; peptides must be transported and loaded onto intact human leukocyte antigen class I (HLA-I) molecules; professional antigen-presenting cells must prime and sustain an appropriate T-cell repertoire; and malignant cells must display the cognate peptide-HLA complex at sufficient density for recognition. Clinical observations in dMMR endometrial cancer already suggest that high potential antigen load does not eliminate downstream escape: resistance-associated alterations and loss of major histocompatibility complex class I (MHC-I) can occur in tumors otherwise considered immunogenic [6,7,8].

We conducted a structured, mechanism-led search of PubMed/MEDLINE. Search concepts combined cervical, endometrial, ovarian, or gynecologic cancer with terms related to antigen presentation, HLA or MHC, B2M, antigen-processing machinery, immunopeptidomics, dendritic cells, cross-presentation, interferon signaling, immune escape, and checkpoint resistance. Peer-reviewed human pathology, treatment-linked, immunopeptidomic, and mechanistic studies were prioritized. Evidence was classified as mechanistic, prognostic, treatment-associated, or prospectively validated, and pan-cancer or experimental-model studies were included only when gynecologic evidence was insufficient.

**Figure 1:**
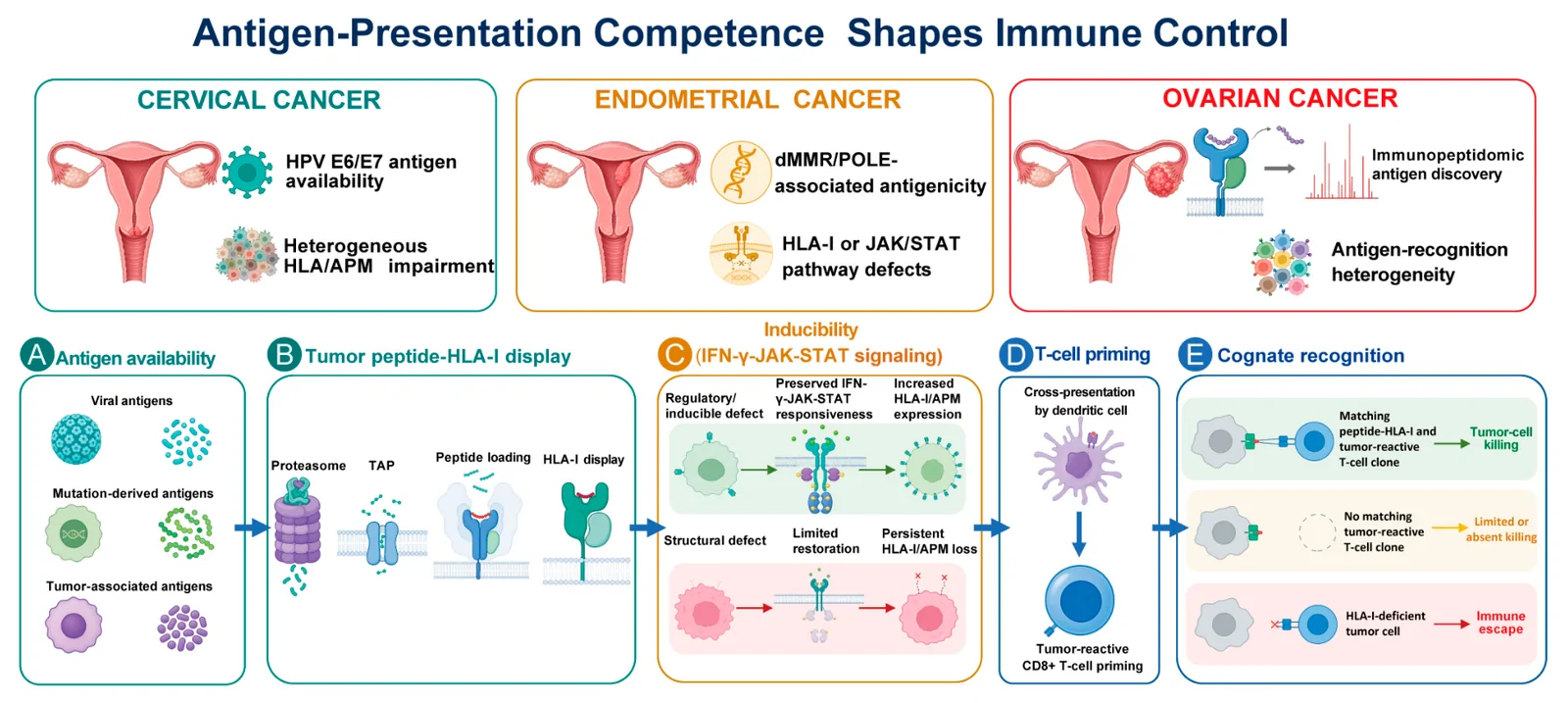
Components of antigen-presentation competence in gynecologic cancers. The upper row contrasts disease contexts; HPV-associated antigens apply to HPV-positive cervical cancers. (**A**) Antigen sources include viral, mutation-derived, and tumor-associated proteins. (**B**) Proteasomal processing generates peptides, TAP transports peptides into the endoplasmic reticulum, and the peptide-loading machinery assembles peptide-HLA-I complexes for surface display. (**C**) IFN-γ-JAK-STAT signaling regulates inducibility; expression recovery requires a sufficiently intact pathway and does not by itself prove restored cognate recognition. (**D**) Dendritic cells acquire tumor-derived material and cross-present it, with costimulation, to prime tumor-reactive CD8+ T cells. (**E**) Effector T cells recognize matching peptide-HLA-I complexes on malignant cells. The horizontal arrows organize related modules, not a strict temporal sequence: direct tumor presentation and professional cross-presentation are distinct routes that converge on recognition, while inducibility regulates several upstream components. The illustrated failure states limit classical CD8-mediated killing but do not establish complete absence of alternative immune control. APM, antigen-processing machinery; dMMR, mismatch-repair deficiency; HLA-I, human leukocyte antigen class I; IFN, interferon; JAK, Janus kinase; POLE, DNA polymerase epsilon; STAT, signal transducer and activator of transcription; TAP, transporter associated with antigen processing.

**Figure 2:**
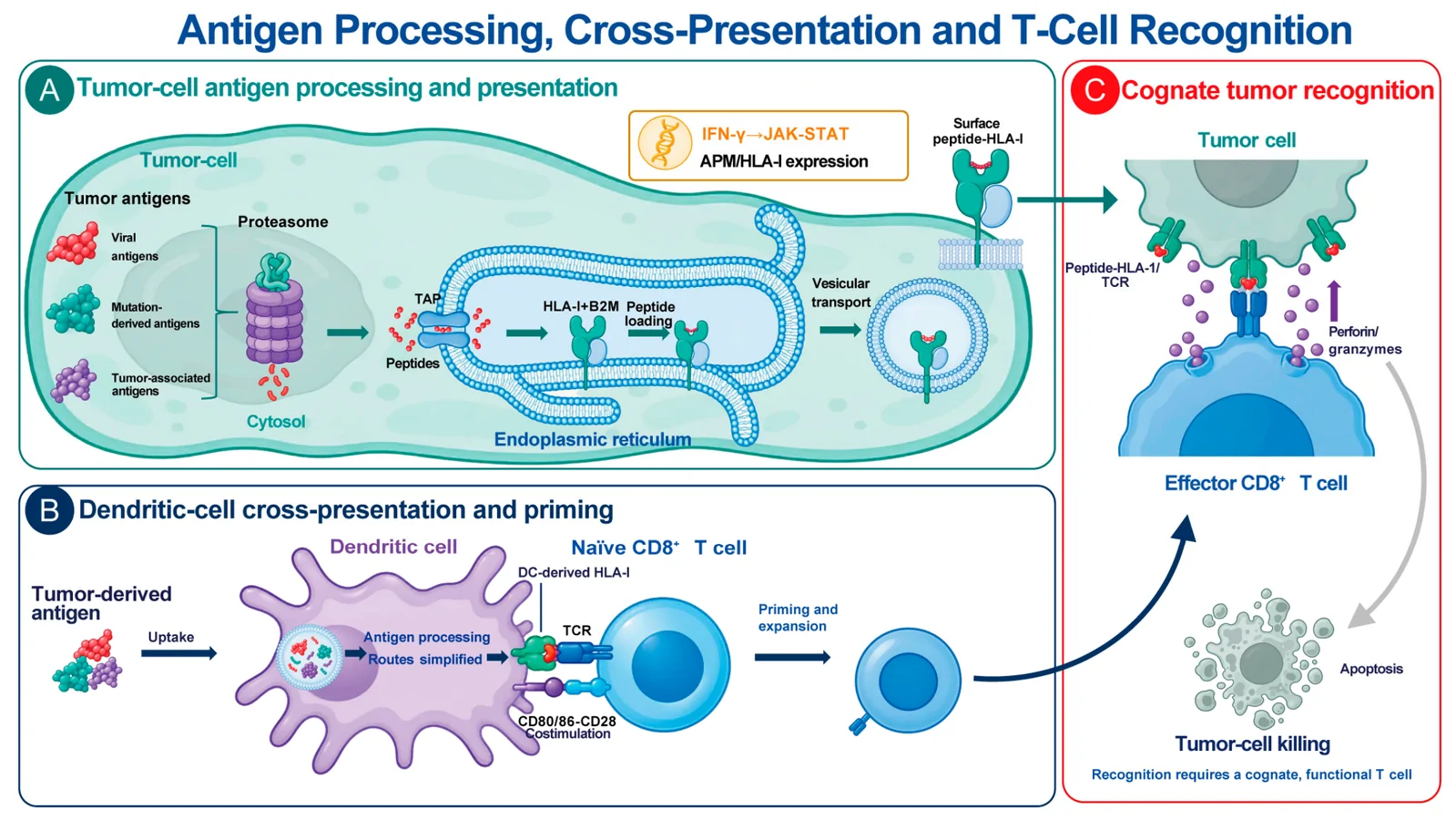
Antigen processing, cross-presentation and T-cell recognition. (**A**) Tumor-cell proteins, including viral, mutation-derived and tumor-associated antigens, are degraded by the proteasome. Peptides enter the endoplasmic reticulum through TAP and are loaded onto HLA class I molecules associated with β2-microglobulin before trafficking to the cell surface. IFN-γ–JAK–STAT signaling regulates antigen-processing machinery and HLA-I expression. (**B**) Dendritic cells take up tumor-derived antigens and cross-present processed peptides on their own HLA-I molecules. Cognate TCR engagement, together with CD80/86–CD28 costimulation, contributes to the priming and expansion of naïve CD8+ T cells. (**C**) Effector CD8+ T cells recognize cognate peptide–HLA-I complexes on tumor cells and can direct perforin/granzyme-mediated cytotoxicity toward the contacted target, leading to apoptosis. The panels show a simplified functional sequence rather than molecular stoichiometry or all required signals. Alternative cross-presentation routes, peptide-loading chaperones and intermediate trafficking compartments are omitted. Antigen presentation alone does not ensure effective tumor-cell killing. APM, antigen-processing machinery; B2M, β2-microglobulin; HLA-I, human leukocyte antigen class I; IFN-γ, interferon-γ; JAK, Janus kinase; STAT, signal transducer and activator of transcription; TAP, transporter associated with antigen processing; TCR, T-cell receptor.

**Figure 3:**
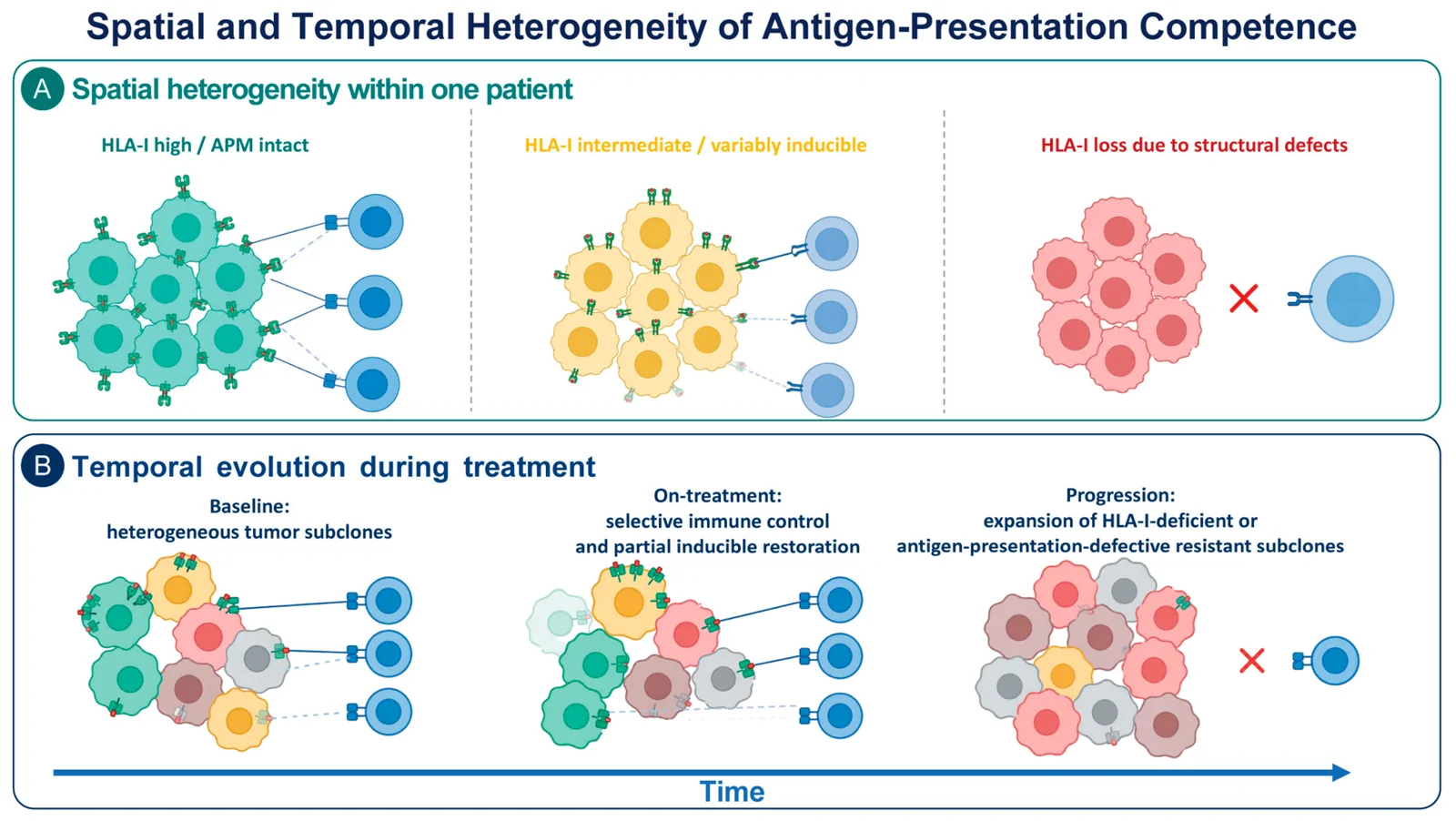
Spatial and temporal heterogeneity of antigen-presentation competence. (**A**) Spatial heterogeneity within one patient is illustrated as HLA-I-high/APM-intact, HLA-I-intermediate/variably inducible, and HLA-I loss due to structural defects, with possible corresponding differences in peptide-HLA-I display and tumor-reactive CD8+ T-cell recognition. (**B**) During treatment, heterogeneous tumor subclones may undergo selective immune control and partial inducible restoration, followed by expansion of HLA-I-deficient or antigen-presentation-defective resistant subclones at progression. These are illustrative trajectories rather than obligatory treatment outcomes; low HLA-I staining alone cannot identify a structural lesion, establish reversibility, or measure T-cell function. APM, antigen-processing machinery; HLA-I, human leukocyte antigen class I.

Antigen presentation denotes the cellular processing and display of peptides bound to HLA molecules. Here, antigen-presentation competence denotes a broader, operational property of the tumor–immune ecosystem: whether antigen supply, malignant-cell display, professional priming, and the cognate T-cell repertoire can jointly support recognition of malignant cells. This expansion is necessary because dendritic cells can prime T cells against an antigen that the tumor fails to display, whereas intact tumor display can coexist with inadequate priming. We organize competence into five domains: antigen availability; tumor-cell peptide-HLA-I display; inducibility of antigen-processing and interferon-response pathways; professional cross-presentation and T-cell priming; and the spatial and temporal integrity of cognate tumor recognition. Recognition is the functional endpoint of this framework, not itself an antigen-presentation event. Each domain should be reported separately as preserved, impaired, heterogeneous, inducible, or unmeasured. This modular profile is neither a scalar score nor an established clinical assay. Its boundary is cognate recognition; trafficking, immunosuppression, and effector dysfunction are considered where they prevent that endpoint, without attempting to encompass the entire cancer-immunity cycle. Figure 1 summarizes the component pathways, Figure 2 details tumor-cell antigen processing, dendritic-cell cross-presentation and T-cell recognition, and Figure 3 illustrates how limiting defects may differ among lesions and treatment time points.

## 2. The Antigen-Presentation Continuum

### 2.1. Antigen Availability Is Not Antigen Presentation

The antigenic starting point differs among gynecologic cancers. HPV-positive cervical cancer provides virally encoded targets, particularly human papillomavirus (HPV) E6 and E7, alongside somatic alterations [9]. Endometrial cancers with POLE mutations or dMMR can generate abundant mutation-derived candidates [10,11]. Epithelial ovarian cancers generally carry a lower average mutation burden but can express cancer-testis antigens and large tumor-associated proteins such as MUC16 and mesothelin [12]. These differences affect antigen supply; they do not determine whether any specific peptide is displayed or recognized.

Computational prediction illustrates the distinction. An altered DNA sequence must be transcribed and translated, and its product must be processed into a suitable peptide. The peptide must then enter the endoplasmic reticulum, bind an individual HLA allele, and persist at the cell surface. Each step is selective. Cervical genomic analyses can identify candidate neo-antigens and associations with HPV-regulated immune activity, but prediction remains upstream of direct presentation [9]. Conversely, an antigen need not arise from a conventional coding mutation. Non-canonical translation, alternative reading frames, and abnormal processing can generate peptides missed by standard exome-based pipelines.

HPV status further separates antigen sources within cervical cancer. HPV-negative disease should not be assigned E6/E7 targets; its candidate antigens instead require assessment of somatic alterations and aberrantly expressed host proteins. TCGA identified a predominantly HPV-negative, endometrial-like molecular group, emphasizing that anatomic site alone does not define a uniform antigenic context [13]. In a retrospective TCGA immune analysis, HPV-negative tumors showed lower lymphocyte-associated signals than HPV α9 (HPV16-like) and α7 (HPV18-like) tumors despite a higher predicted neo-antigen burden [14]. These are genomic and transcriptomic observations, not measurements of tumor-cell peptide display or patient-specific recognition. HPV genotype, viral transcription, and host HLA genotype should therefore be recorded separately: detection of viral DNA does not quantify E6/E7 expression or demonstrate a display of a particular epitope. Reported differences between α9 and α7 groups do not yet support a genotype-specific competence threshold [9,13,14].

Even in HPV-positive disease, viral antigenicity can coexist with HLA allelic loss or APM impairment, reducing the set of viral peptides visible to effector T cells [15,16,17,18]. Immune exclusion can prevent contact with otherwise recognizable malignant cells, while dysfunctional T cells and suppressive myeloid signals can limit responses despite persistent antigen expression [14,19,20,21]. Thus, HPV positivity describes an antigenic opportunity; it cannot establish effective immune recognition, and antigen expression, tumor-cell display, tissue access, and T-cell function require separate assessment.

Ovarian cancer provides particularly direct gynecologic evidence for this gap. Direct HLA ligandome analysis identified naturally presented class I and class II peptides derived from ovarian cancer-associated proteins, including MUC16 and mesothelin, and showed an imperfect relationship between transcript abundance and peptide display [12]. More recent studies expanded this landscape to immunogenic cryptic peptides and shared proteogenomically defined candidates [22,23]. A relatively low TMB, then, need not imply a uniformly sparse antigenic surface. However, direct detection closes only the gap between prediction and presentation. It does not prove that a peptide is tumor exclusive, sufficiently abundant across all disease sites, or recognized by a functional autologous T-cell clone. An integrated ovarian cancer case study demonstrated discordance among mutation detection, predicted binding, MHC-I peptidomics, and patient T-cell responses [24]. Antigen availability, presentation, and immunogenicity must therefore remain separate analytical levels [25].

The ovarian studies support complementary, rather than interchangeable, inferences. Ligandome studies establish natural peptide display in the sampled material [12,22,23]. In the single-patient study by Dao and colleagues, several candidate epitopes elicited responses from healthy-donor T cells but not from the patient’s tested ascites or peripheral-blood T cells, illustrating why donor immunogenicity does not demonstrate an available autologous repertoire [24]. In contrast, multiregion immune-receptor and genomic analyses associate local immune infiltration with neo-antigen depletion, HLA loss, and tumor-clone distribution; they do not assign cognate specificity to every tracked T-cell clone [26]. Together, these studies motivate region-matched measurements of peptide display and autologous tumor-cell recognition, rather than inferring the latter from either mass spectrometry or T-cell receptor (TCR) clonality alone.

### 2.2. Tumor-Cell Visibility Depends on the HLA-I Antigen-Processing Machinery

Classical tumor-cell recognition by CD8+ T cells requires coordinated antigen-processing machinery (APM). Proteasomal and immunoproteasomal cleavage generates peptide substrates. The transporter associated with antigen processing 1/2 (TAP1/2) moves these peptides into the endoplasmic reticulum, where HLA-I heavy chains associate with β2-microglobulin (B2M), and tapasin and other loading-complex components support peptide loading and stable complex assembly before surface transport. Interferon (IFN)-Janus kinase (JAK)-signal transducer and activator of transcription (STAT) signaling and the transcriptional regulator NLRC5 regulate multiple elements of this pathway. A low-presentation phenotype can thus reflect a structural lesion, allele-specific loss, impaired peptide loading, epigenetic repression, or inadequate inducible signaling [11,27].

Cervical carcinoma studies have documented abnormalities at several levels. Individual HLA-A and HLA-B alleles can be downregulated in relation to TAP1 expression [15], while broader analyses link antigen-processing machinery defects with clinicopathological outcome [16]. In a small molecular series, 15 of 16 cervical carcinomas had reduced expression of at least one antigen-processing component; several defects in cell lines were partly restored by IFN-γ, supporting regulatory suppression in at least some settings [17]. HLA-I allelic loss during cervical neoplastic progression provides complementary evidence of structural immune selection [18]. Although these studies span different eras and assays, they collectively suggest that the presence of HPV antigens does not guarantee intact tumor-cell display.

Endometrial cancer provides a relatively direct bridge from pathology to immunotherapy outcome. In a series of 76 carcinomas, 42% showed subclonal or diffuse MHC-I loss, including 46% of dMMR tumors [7]. Two features of this result matter here. First, high potential neo-antigen load can coexist with a downstream recognition defect. Second, subclonal loss makes a binary positive–negative designation dependent on sampling. Genomic observations in resistant dMMR tumors further implicate antigen-presentation escape [6]. A prospective neoadjuvant phase I study offers treatment-linked tissue context, although it was not designed to validate a presentation biomarker [1]. More recently, a translational cohort of 84 patients treated with immune-checkpoint inhibitors (ICIs) associated MHC-I expression with improved progression-free survival and showed that the consequences of JAK1 pathogenic variants depended on allelic and signaling context [2]. The signal warrants further validation, although neither an invariant resistance rule nor an independently validated score has been established.

Ovarian carcinoma likewise shows heterogeneity in presentation machinery. HLA-I antigen-processing components and intratumoral T-cell infiltration have independent prognostic associations [28], and metastatic selection of HLA-A2 haplotype loss has been documented in the setting of HLA-A2-restricted immunity [29]. A multiregion study of 212 samples from 38 patients with high-grade serous ovarian cancer integrated whole-genome sequencing, immune-receptor profiling, and spatial analyses. It linked local immune pressure with neo-antigen depletion, HLA-I loss of heterozygosity (LOH), and divergent tumor–immune clonal dynamics across disease sites [26]. Presentation competence can therefore differ among lesions in one patient, a limitation that no single-tissue estimate can capture.

### 2.3. Professional Priming Is Not Tumor-Cell Recognition

Tumor-cell display represents only one side of the interaction. Conventional type 1 dendritic cells (cDC1 cells) acquire tumor antigens and cross-present them to CD8+ T cells. Experimental studies show that Batf3-lineage dendritic cells support effector T-cell trafficking, that natural killer (NK) cell-derived signals can recruit cDC1 cells, and that cDC1 cells maintain proliferative tumor-antigen-specific T-cell reservoirs in draining lymph nodes [30,31,32]. These findings provide mechanistic support, although most supporting experiments used non-gynecologic models. Mature dendritic-cell density and favorable immune organization in ovarian carcinoma provide human corroboration without proving endogenous cDC1 cross-presentation in individual patients [19,20,33].

Antigen uptake, cross-presentation, and priming are distinct experimental steps. Uptake demonstrates acquisition of tumor material; cross-presentation requires its processing and display on the dendritic cell’s own HLA-I; productive priming additionally requires appropriate costimulation and differentiation signals for naïve cognate T cells. Tumor HLA-I staining measures none of these dendritic-cell functions. Conversely, peptide-pulsed dendritic cells can test T-cell responsiveness while bypassing uptake and antigen processing, so vaccine-induced responses cannot be assumed to demonstrate endogenous cross-presentation. Mechanistic assays should therefore use tumor-derived material and antigen-specific T-cell readouts, with controls for HLA restriction and processing dependence [30,31,32,34,35].

Separating priming from target recognition clarifies apparently paradoxical states. Preserved cross-presentation may generate antigen-specific T cells even when malignant cells have lost the relevant HLA molecule. Conversely, tumor cells may display a potentially recognizable complex while insufficient priming leaves the relevant repertoire absent or functionally weak. A pilot cervical cancer study of HPV E7-loaded dendritic cells illustrates the distinction: cellular immune responses occurred in a subset, but severe or complete tumor HLA loss was present in three of four available biopsies and no objective clinical responses were observed [36]. A small endometrial dendritic-cell vaccination study likewise demonstrated antigen-specific responses in only a subset of treated patients [37]. Neither trial validates a cDC1 biomarker. Both nevertheless illustrate why immune-response induction and malignant-cell recognition require separate measurements.

## 3. From Proxy Biomarkers to Functional Competence

### 3.1. Current Biomarkers Interrogate Different Levels

The limitation lies less in the biomarkers themselves than in treating their distinct outputs as interchangeable. dMMR, POLE mutation, and TMB estimate altered-peptide-generating potential. PD-L1 indicates an inhibitory ligand and may reflect interferon exposure. CD8 density indicates recruitment or prior activation, but not necessarily tumor specificity or effective cytotoxicity. HLA-I immunohistochemistry can reveal loss or spatial heterogeneity, but not the identity, abundance, or immunogenicity of displayed peptides. RNA-based antigen-processing signatures measure transcription rather than allele-specific structural integrity, protein assembly, or surface display. Immunopeptidomics directly samples HLA-associated peptides, but has incomplete sensitivity, depends on HLA polymorphism and tissue input, and does not by itself establish T-cell recognition or whole-patient spatial coverage [38,39,40,41].

A more informative approach treats these measurements as complementary modules rather than universal alternatives competing for rank. A tumor may be TMB-high but presentation-deficient, HLA-I-positive but poor in relevant peptides, peptide-positive but deficient in priming, or immune infiltrated yet dominated by irrelevant or dysfunctional clones. Table 1 summarizes what representative measurements establish, their inferential status, and the conclusions they cannot support. Table 2 compares the same biological dimensions across the three diseases and identifies differences in evidence maturity. The framework is most useful when it makes the permitted inference from each measurement explicit.

### 3.2. Structural and Regulatory Defects Form a Provisional Spectrum

The established distinction between “hard” and “soft” HLA-I presentation defects provides one mechanistic axis within the broader competence profile; it is not synonymous with the full framework. Hard lesions include biallelic B2M inactivation, HLA-region loss of heterozygosity, destructive HLA heavy-chain abnormalities, and functionally disabling lesions in essential interferon signaling. Such lesions may be difficult to reverse within the malignant clone, depending on lesion type and clonality. HLA LOH may remove selected alleles while preserving others, and a JAK-pathway alteration requires allelic and functional interpretation rather than automatic classification as complete signaling failure [2]. Soft defects include epigenetic or transcriptional repression of HLA-I, TAP, tapasin, or related components when the underlying pathway remains inducible. Interferon exposure can restore selected cervical antigen-processing abnormalities in model systems [17] and remodel the ovarian cancer HLA ligandome [46]. The competence framework adds the domains of antigen availability, professional priming, cognate recognition, and spatial/temporal clonality, so two tumors with similar HLA-I expression may still occupy different functional states.

The hard–soft terminology does not define a clinical binary. Baseline low expression does not establish that a pathway is reversible, while a genomic alteration does not reveal its clonality or residual function. The cervical chemoradiotherapy setting illustrates the problem: paired biopsies before and during treatment showed heterogeneous changes in CD8 infiltration, interferon regulatory factor 1 (IRF1), and PD-L1 rather than a uniform inflammatory conversion [42]. Functional perturbation, longitudinal sampling, or direct demonstration of restored peptide display are therefore needed before an individual lesion is labeled reversible. Hard and soft are best treated as ends of a mechanistic spectrum.

A practical distinction requires orthogonal evidence. Tumor-cell-resolved HLA/B2M staining should be paired with allele-specific genomic and copy-number analysis to establish whether a candidate structural lesion is present and how widely it is distributed. Ex vivo IFN-γ challenge, with proximal signaling and HLA/APM readouts, can test inducibility; peptide-HLA recovery and HLA-restricted recognition provide stronger functional rescue endpoints. Matched samples can distinguish expression changes within persistent clones from the expansion of a different clone. Neither a single negative stain nor an isolated mutation is sufficient, and unsuccessful rescue under one experimental condition does not by itself prove irreversibility [2,17,46].

### 3.3. Spatial and Temporal Heterogeneity Limits Static Classification

Subclonal endometrial MHC-I loss [7], metastatic ovarian HLA haplotype loss [29], and multiregion HLA-I LOH [26] all challenge patient-level classification from one specimen. Therapy adds a temporal dimension. In cervical cancer, interferon-linked immune changes during chemoradiotherapy varied between patients [42], and serial systemic monitoring found impaired antigen-presenting-cell stimulatory capacity during conventional treatment [55]. The latter finding is blood-based and cannot substitute for tumor assessment, but it underscores that priming capacity also changes over time [56].

Pan-cancer trial-biopsy data provide indirect support for dynamic measurement. In an analysis of 213 baseline and 116 on-treatment biopsies from metastatic lesions, B2M and HLA-A expression varied by tumor type and disease context. High on-treatment B2M expression correlated with response more consistently than baseline expression, and low baseline B2M often increased after immunotherapy [43]. Although these observations are not specific to gynecologic cancer, they illustrate how a fixed baseline score can conflate irreversible clonal loss with inducible suppression and obscure their distinct biological implications.

Spatial uncertainty has disease-specific manifestations: heterogeneous HLA/APM abnormalities in cervical cancer, subclonal MHC-I loss in endometrial cancer, and divergent HLA and immune-receptor patterns among ovarian lesions [7,15,16,17,18,26,28,29,45]. A sample with preserved presentation cannot exclude a deficient region elsewhere; conversely, a deficient core cannot establish uniform patient-level loss. Chemotherapy and radiotherapy may change antigen release, inflammatory signaling, and antigen-presenting-cell function; checkpoint blockade may change local IFN exposure and select immune-evasive clones; targeted therapies may modulate presentation pathways in an agent-dependent manner [42,43,55,56,57,58,59,60]. These processes should not be collapsed into an assumed increase in competence. Serial measurements must distinguish regulatory change, altered cell composition, and clonal selection. Competence is therefore a candidate dynamic biomarker framework, with each result indexed to lesion, treatment, and sampling time.

A clinically credible competence assessment should record spatial pattern and time point, not merely marker intensity. Three implementation tiers can separate scientific ambition from deployability. A discovery tier can combine multiregion genomics, HLA typing, loss-of-heterozygosity analysis, functional interferon perturbation, T-cell receptor sequencing, spatial profiling, and immunopeptidomics to identify limiting gates. A translational-trial tier can use paired baseline and on-treatment biopsies with multiplex pathology, targeted DNA/RNA profiling, selected functional assays, and banked tissue for deeper analysis. A routine-clinical tier, if prospectively validated, would need a small pathology-accessible panel with defined tissue requirements, interlaboratory reproducibility, clinically compatible turnaround, and an explicit action linked to each result. Because sampling error could falsely label a heterogeneous tumor as presentation-incompetent, tissue adequacy, lesion selection, repeat testing, and the potential harm of false-negative treatment exclusion must be built into validation. The goal is to resolve distinct failure states, not to maximize assay complexity for every patient.

### 3.4. MHC-I Loss Impairs Classical Recognition but Does Not Prove Complete Immune Silence

Loss of tumor-cell MHC-I directly constrains cognate CD8+ T-cell recognition. MHC-I loss is therefore biologically plausible as a resistance mechanism. Yet converting this mechanism into a deterministic clinical rule ignores alternative immunity and lesion heterogeneity. In the 84-patient endometrial cohort, some tumors with JAK1 alterations had unexpectedly favorable outcomes, and NK-cell markers were associated with survival in a subset [2]. In B2M-deficient murine tumors, CD4+ T cells and NK cells mediated checkpoint-associated control in selected models; analysis of melanoma biopsies also suggested interactions between partial B2M loss and activated NK infiltration [61].

Compensation is possible in these settings, but its reliability remains uncertain. The effects differed between models, and the human evidence is not gynecologic-specific. MHC-I loss should therefore be described asymmetrically: impairment of the classical CD8 route is the direct consequence, whereas NK- or CD4-mediated control is conditional. This distinction prevents both overcalling universal resistance and implying that alternative immunity routinely neutralizes HLA loss.

### 3.5. Prognostic Association Is Not Predictive Validation

Much of the gynecologic evidence remains prognostic, descriptive, or mechanistic. Ovarian antigen-processing machinery and T-cell studies associate expression patterns with survival [28,62], but do not demonstrate differential benefit from immune-checkpoint therapy. Cervical studies establish frequent HLA/APM abnormalities and treatment-induced immune heterogeneity, whereas direct evidence that these measurements select patients for checkpoint inhibition remains sparse. Endometrial cancer offers stronger treatment-linked observations [1,2,6], but no study has prospectively validated a composite antigen-presentation profile or demonstrated incremental predictive performance over mismatch-repair status, polymerase epsilon, PD-L1, and TMB together.

Claim strength should follow this evidence hierarchy. Biological relevance and prognostic association do not establish treatment prediction. Associations within an immune-checkpoint-treated cohort are more informative, but remain vulnerable to confounding unless treatment interaction, independent validation, and predefined thresholds are demonstrated. The appropriate next step is therefore prospective testing of antigen-presentation competence as a candidate model alongside, rather than in place of, established biomarkers.

### 3.6. Broader Evidence Strengthens the Framework but Not Its Clinical Validation

Additional cervical studies refine the biological modules rather than alter their clinical status. Combined allele-specific phenotyping and genotyping demonstrate frequent, heterogeneous HLA-I loss arising through several genetic mechanisms, while single-cell studies show that radiochemotherapy and histologic context reshape antigen-presentation and immune programs [45,63,64]. A cervical cancer model also links tumor-associated macrophage metabolism to impaired antigen cross-presentation, identifying a myeloid route by which the local microenvironment may constrain T-cell priming [21]. Together, these findings localize several potential failure points but do not establish a validated patient-selection rule.

In endometrial cancer, molecularly distinct causes of mismatch-repair deficiency are associated with different immune states and response patterns during PD-1 blockade [10]. Proteogenomic characterization provides a broader substrate for connecting genomic subtype, protein expression, and immune signaling [11], whereas experimental LATS1/2 loss has been linked to reduced MHC-I expression and immune evasion [65]. These data support a layered model in which antigen-generating potential, signaling competence, and surface presentation vary independently, and they caution against treating all mismatch-repair-deficient tumors as biologically equivalent.

Ovarian and cross-gynecologic studies likewise broaden the antigen repertoire beyond conventional mutation-derived candidates. Catalogs of gynecologic T-cell targets and ovarian studies of TP53 hotspot neo-antigens, patient-specific neo-antigens, endogenous retroviral epitopes, HLA-presented histone deacetylase-derived peptides, and multi-omics-defined autoantigens demonstrate multiple routes to recognizable targets [47,48,49,50,51,52]. Early translational studies of personalized neo-antigen-expanded T cells and T-cell receptors directed against shared antigens support biological feasibility [53,54], while cancer-testis antigen reviews summarize additional candidate targets [66]. The collective contribution is evidence of antigen diversity and tractability, not population-level therapeutic efficacy.

Tumor-intrinsic regulatory networks provide additional contexts for testing presentation phenotypes. Huang and colleagues identified an AC093895.1–miR-1253–SOX4 positive-feedback network that promotes ovarian cancer growth and metastasis [67]. Zhou and colleagues combined network pharmacology with HeLa-cell experiments and linked naringenin exposure to reduced migration and invasion and suppression of EGFR/PI3K/AKT/mTOR signaling [68]. These studies establish aspects of tumor regulation and provide candidate perturbations for future immune-recognition experiments. They do not directly demonstrate altered peptide-HLA display, dendritic-cell priming, or cognate T-cell killing. Connecting these pathways to competence will require measuring those endpoints alongside tumor viability, so that direct cytotoxicity is not mistaken for improved immune recognition.

## 4. A Testable Translational Agenda

### 4.1. Build Modular Measurements and Avoid Circularity

A first-generation assessment should preserve the five domains as an interpretable state vector. Antigen availability could be represented by viral-antigen status, dMMR/MSI/POLE/TMB context, expressed candidate antigens, and, in research settings, detected HLA ligands. Tumor display could combine spatial HLA-I/B2M/APM protein expression with HLA typing and allele-specific genomic loss. Inducibility could be measured through paired treatment samples or functional interferon-response assays. Professional priming could combine cDC1 state, localization, cross-presentation readouts, and tumor-draining-node or T-cell-repertoire measures. Cognate recognition and clonality could incorporate spatial CD8+ T-cell-tumor proximity, antigen-specific T-cell assays, T-cell receptor tracking, and multisite concordance. Each domain should output a categorical state with an uncertainty flag and a record of missing measurements. These domains should not be collapsed prematurely into a weighted number because the weights, compensation rules, and clinically meaningful cutoffs are unknown.

Assay selection must also account for dendritic-cell heterogeneity and cross-presentation biology. Single-cell reference maps distinguish conserved human and murine dendritic-cell states [69], while WDFY4- and DNGR-1-dependent studies identify specific requirements for tumor-antigen cross-presentation [34,35]. More recent work describes stimulatory cDC1 subsets and metabolic control of cDC1 antitumor function [70,71]. Collectively, these studies favor functional or state-resolved measurement over reliance on a single lineage marker. However, the proposed readouts remain predominantly experimental and should inform assay design rather than define clinical cutoffs.

Cognate recognition should be operationalized as an antigen- and HLA-dependent T-cell response to a defined target. TCR sequencing tracks repertoire composition and expansion but does not by itself establish antigen specificity; peptide-HLA multimer binding identifies compatible T cells but does not demonstrate activation or killing. Functional evidence can include TCR-reporter activation, activation-marker induction, antigen-dependent cytokine production, degranulation, and direct cytotoxicity against autologous tumor cells. Peptide-pulsed targets test epitope reactivity but bypass endogenous tumor processing. Stronger evidence of malignant-cell visibility combines recognition of unpulsed tumor targets with HLA blockade or genetic disruption and matched antigen-positive and antigen-negative controls [19,24,38]. Autologous organoid–T-cell coculture can connect activation with tumor killing, as demonstrated in colorectal and lung cancers; this is a transferable experimental approach, not gynecologic clinical validation [44]. Culture selection, IFN pretreatment, and loss of stromal or myeloid compartments must be reported because they can change the state being measured.

A central validation question is: does a prespecified competence profile provide reproducible predictive information beyond dMMR/MSI, POLE, PD-L1, and TMB? A prospective study should define the profile as the index test before outcome analysis, collect baseline and, where feasible, early on-treatment tissue, and use objective response or a prespecified time-to-event endpoint under a defined therapy. Established biomarkers should form the comparator model; each competence domain should then be added without using downstream response-like features in its definition. Multilevel models should account for lesions nested within patients, while discrimination, calibration, reclassification, decision-curve value, and interlaboratory reproducibility should be assessed in a development cohort and confirmed in an external cohort. Clinical utility would be unsupported if the profile failed to improve the comparator model or performed inconsistently across laboratories or lesions. It would also be unsupported if it could not represent subclonality or inform a management decision that improves outcomes. A negative result would reject the proposed clinical use even if the biological components remain valid.

### 4.2. Use Paired and Multisite Specimens

Neoadjuvant and window-of-opportunity studies are particularly suitable because they can connect baseline state, early pharmacodynamic change, surgical pathology, and longer-term outcome. An informative design would collect pretreatment tumor, an on-treatment specimen where feasible, definitive surgical tissue, and blood, with metastatic or draining-node samples when obtained for clinical reasons. Spatial HLA/APM pathology could be integrated with genomic lesions, functional interferon responsiveness, cDC1 organization, T-cell receptor dynamics, and direct peptide presentation in nested cohorts.

Emerging dendritic-cell engineering approaches offer experimental tools for testing causality in these designs. Cytokine-armed dendritic-cell progenitors, in vivo dendritic-cell reprogramming, and dendritic-T-cell engagers augment antitumor immunity in models [72,73,74]. Radiotherapy provides another perturbation whose effect depends on exposure: induction of three-prime repair exonuclease 1 (TREX1) can limit radiation-driven tumor immunogenicity [75]. Embedded in window studies, such interventions could help identify the presentation gate that limits response rather than being interpreted as established treatment pairings [56,76].

Longitudinal analysis can separate three phenomena that look similar in a static assay: a baseline structural defect, therapy-induced restoration, and acquired immune escape. Multisite sampling can determine whether a minor presentation-deficient clone at diagnosis becomes dominant at progression. Although this design is demanding, it directly addresses the measurement limitations revealed by current pathology and multiregion studies.

### 4.3. Match Experimental Strategies to the Lesion

Mechanistic classification can organize therapeutic hypotheses without becoming a treatment algorithm. Low HLA/APM expression with preserved inducibility could be studied using radiotherapy, interferon-pathway or innate-immune activation, or epigenetic modulation. Inadequate dendritic-cell recruitment or activation could motivate investigation of FMS-like tyrosine kinase 3 ligand, CD40 agonism, or stimulator of interferon genes (STING)-pathway activation. Structural B2M or HLA loss could motivate relatively HLA-independent approaches, including selected NK-cell, chimeric antigen receptor, or bispecific platforms. When tumor display is preserved but the relevant T-cell repertoire is insufficient, vaccination or T-cell receptor-engineered approaches may be more mechanistically coherent.

The wider checkpoint-resistance literature supplies cautionary precedents. Acquired defects in HLA processing, JAK signaling, or B2M have been described after PD-1 blockade in lung cancer and melanoma [57,58,59], while therapy-induced regulation of HLA/APM components varies across chemotherapy, radiation, and targeted agents [60]. Contemporary models of tumor immunity further separate early lymph-node activation from intratumoral effector differentiation [77]. These observations support a treatment- and context-dependent framework, with broad HLA-I surveillance taxonomies providing orientation while primary gynecologic evidence retains priority [78].

Evidence for these pairings remains predominantly preclinical, pan-cancer, or exploratory. Reversible expression alone does not establish restoration of an immunogenic ligandome or clinical sensitivity. These interventions are therefore best deployed as perturbations that test the framework and its limiting gates.

## 5. Discussion and Conclusions

Antigen-presentation competence offers a way to connect tumor antigenicity with effective immune recognition while making the intermediate failure points explicit. Its evidentiary basis, however, differs sharply by disease and by domain. Cervical cancer demonstrates HPV-associated selection and multiple HLA/APM abnormalities; endometrial cancer provides relatively direct treatment-linked evidence; and ovarian cancer reveals the gap between antigen prediction, direct presentation, and spatially heterogeneous immune editing. Across the three diseases, the central lesson is that mutation burden, RNA expression, protein staining, peptide display, T-cell priming, and tumor recognition are related but non-interchangeable measurements. Figure 1, Figure 2 and Figure 3 provide the accompanying conceptual summaries.

The framework also has clear limits. No prospectively validated antigen-presentation competence profile exists, and incremental predictive value beyond established biomarkers has not been demonstrated. Despite structured searching and citation verification, this integrative, non-systematic review remains vulnerable to study-selection and publication bias. Assays differ in antibodies, thresholds, HLA alleles, tissue input, platforms, treatment timing, and spatial sampling, limiting comparison across studies and transfer between cervical, endometrial, and ovarian cancers. Hard and soft defects remain a provisional spectrum, alternative immune control after MHC-I loss is context-dependent, and much of the cDC1, engineering, and acquired-resistance evidence is preclinical or imported from other tumor types. These constraints do not support a clinical cutoff or treatment rule; established biomarkers should retain their current roles while the modular profile undergoes prospective testing.

Three evidentiary levels should remain explicit. Biological plausibility follows from the established requirements for antigen processing, HLA display, priming, and recognition. Experimental validation requires showing that a specified defect limits these functions and that its perturbation changes the relevant endpoint. Clinical utility requires more: standardized assays and tissue requirements, reproducibility across laboratories and tumor regions, interpretable longitudinal changes, external cohort validation, and prospective evidence that using the profile improves treatment selection or patient outcomes beyond established biomarkers. A prespecified assay that predicts outcome only within a treated cohort has not yet demonstrated this utility. Antigen-presentation competence should therefore be evaluated as a falsifiable framework whose clinical value depends on reproducible incremental information and a beneficial management consequence.

## Figures and Tables

**Table 1:** Evidence and measurement boundaries across the antigen-presentation continuum.

Cancer/Context	Component or Assay	Representative Evidence	Inferential Status	What it Establishes	What It Cannot Establish
Cervical	HLA allelic loss and APM expression	Allele-specific downregulation, multi-component defects, and HLA loss during progression [15,16,17,18]	Mechanistic/pathological; partly prognostic	Presentation defects occur despite viral-antigen availability	Independent prediction of checkpoint response
Cervical, during treatment	Paired tumor IFN/immune markers	Heterogeneous CD8, IRF1, and PD-L1 changes during chemoradiotherapy [42]	Longitudinal, treatment-associated	Immune state is dynamic and patient-specific	Direct restoration of peptide-HLA display or durable ICI benefit
Endometrial	MHC-I IHC, B2M/JAK1 context	Subclonal/diffuse loss and ICI-treated translational cohorts [1,2,6,7]	Pathological and treatment-associated	Presentation status may explain heterogeneity within dMMR disease	Validated competence profile or deterministic resistance rule
Ovarian	APM expression and HLA loss	Prognostic associations, metastatic haplotype loss, and multiregion HLA-I LOH [26,28,29]	Prognostic/spatial evolutionary	Immune editing and lesion-level heterogeneity	Checkpoint-treatment prediction
Ovarian	Immunopeptidomics	Canonical, shared, and cryptic HLA ligands [12,22,23,24]	Direct presentation evidence	Specific peptides are naturally displayed	Whole-patient coverage or effective T-cell recognition
Pan-cancer mechanistic	cDC1 and draining-node T cells	Recruitment, trafficking, and T-cell maintenance studies [30,31,32]	Preclinical mechanism	Priming is distinct from tumor-cell display	Gynecologic clinical validation
Pan-cancer treatment biopsies	HLA-A/B2M IHC over time	Stage- and treatment-dependent expression [43]	Indirect treatment-associated	Baseline expression may not represent on-treatment state	Cancer-specific thresholds or a gynecologic selection rule
Ovarian examples; broader experimental models	Cognate recognition: TCR sequencing; peptide-HLA multimers; activation/cytokine and killing assays; autologous organoid–T-cell coculture	Patient versus donor reactivity [24]; T-cell specificity [19]; organoid killing in colorectal/lung models [44]	Repertoire/specificity proxies; functional evidence only with target-dependent readouts	Separates clone tracking, epitope binding, activation, and HLA-restricted tumor killing	Sequencing or multimer binding alone does not prove endogenous tumor recognition; culture assays do not establish clinical benefit

**Table 2:** Comparative evidence for antigen-presentation competence across gynecologic cancers.

Dimension	Cervical Cancer	Endometrial Cancer	Ovarian Cancer
Antigen generation	HPV-positive: viral E6/E7; HPV-negative: host-derived candidates. Genotype/expression matter [9,13,14].	dMMR/POLE-associated candidate antigens; molecular subtype affects immune context [10,11].	Canonical and cryptic HLA ligands despite often modest TMB [12,22,23].
Processing machinery	TAP and other APM defects; selected IFN-responsive model defects [15,16,17].	Presentation-pathway abnormalities; genomic/protein context differs by subtype [6,11].	APM expression has prognostic associations; transcript levels do not specify the ligandome [12,28].
HLA expression and integrity	Allele-specific downregulation and genetic HLA loss [15,16,17,18,45].	Subclonal/diffuse MHC-I loss, including dMMR tumors; treatment-associated evidence [2,7].	Haplotype loss and multiregion HLA LOH; direct ligandome evidence [12,26,29].
Interferon signaling	Model inducibility; heterogeneous paired chemoradiotherapy markers [17,42].	JAK1 effects depend on allelic/signaling context [2].	IFN-driven ligandome remodeling in experimental systems [46].
Dendritic-cell function	Small DC-vaccine study separates induced responses from tumor HLA loss [36].	Small DC-vaccination trial; responses in a subset [37].	Mature DC density associated with prognosis; not proof of endogenous cross-presentation [33].
T-cell recognition	HPV targets are candidates; systemic responses do not establish malignant-cell recognition [9,36].	Antigen-specific vaccine responses do not validate a tumor-recognition assay [37].	Patient/donor reactivity discordance and selected antigen-targeting studies [24,47,48,49,50,51,52,53,54].
Spatial heterogeneity	Heterogeneous HLA/APM expression; single-biopsy inference limited [15,16,17,45].	Subclonal MHC-I loss challenges binary classification [7].	Direct multiregion evidence linking immune and malignant clones [26,29].
Experimental and clinical maturity	Predominantly pathology, mechanisms, and exploratory longitudinal evidence; no validated selection profile.	Relatively direct ICI-associated cohorts and early treatment studies; no prospective profile validation [1,2,6].	Strong presentation and spatial evidence; mostly prognostic or exploratory treatment evidence, not ICI prediction.

Evidence maturity refers to the specific domain, not an overall disease ranking. None of the three diseases has a prospectively validated composite competence profile. DC, dendritic cell; ICI, immune-checkpoint inhibitor; LOH, loss of heterozygosity. Other abbreviations are defined in the text.

## Data Availability

No new data were created or analyzed in this study. Data sharing is not applicable to this article.
